# The *Rhinella arenarum* transcriptome: *de novo* assembly, annotation and gene prediction

**DOI:** 10.1038/s41598-020-57961-4

**Published:** 2020-01-23

**Authors:** Danilo Guillermo Ceschin, Natalia Susana Pires, Mariana Noelia Mardirosian, Cecilia Inés Lascano, Andrés Venturino

**Affiliations:** 1 Centro de Investigaciones en Toxicología Ambiental y Agrobiotecnología del Comahue (CITAAC), Consejo Nacional de Investigaciones Científicas y Técnicas (CONICET)-Universidad Nacional del Comahue, Buenos Aires 1400, Neuquén, CP 8300 Neuquén, Argentina; 2 Present Address: Laboratorio de Bioinformática Traslacional, Centro de Investigaciones en Medicina Traslacional Severo Amuchástegui, Instituto Universitario de Ciencias Biomédicas de Córdoba. Av. Naciones Unidas 420, CP 5000 Córdoba, Argentina

**Keywords:** Agroecology, Agroecology, Molecular ecology

## Abstract

The common toad *Rhinella arenarum* is widely distributed in Argentina, where it is utilised as an autochthonous model in ecotoxicological research and environmental toxicology. However, the lack of a reference genome makes molecular assays and gene expression studies difficult to carry out on this non-model species. To address this issue, we performed a genome-wide transcriptome analysis on *R. arenarum* larvae through massive RNA sequencing, followed by *de novo* assembly, annotation, and gene prediction. We obtained 57,407 well-annotated transcripts representing 99.4% of transcriptome completeness (available at http://rhinella.uncoma.edu.ar). We also defined a set of 52,800 high-confidence lncRNA transcripts and demonstrated the reliability of the transcriptome data to perform phylogenetic analysis. Our comprehensive transcriptome analysis of *R. arenarum* represents a valuable resource to perform functional genomic studies and to identify potential molecular biomarkers in ecotoxicological research.

## Introduction

Amphibians are poikilothermic vertebrates with morphological and ecological adaptations that allow them to occupy diverse terrestrial environments associated with humid ecosystems^1,2^. They are the only terrestrial vertebrates that preserve free-living larvae and produce large oocytes with a transparent vitelline membrane that allows for the direct observation of the different stages of embryonic development. These characteristics have been exploited in various research areas such as toxicology, physiology, ecology, and evolution^3–7^. The South American common toad *Rhinella arenarum* [ex. *Bufo arenarum* (Hensel, 1867)] is amply distributed in Argentina and breeds in shallow-water areas such as ponds and ditches^5,8,9^.

Amphibian research models can be easily and inexpensively established. However, only six anuran genomes are available to date: *Pyxicephalus adspersus*^10^, *Nanorana parkeri*^11^, *Rana catesbeiana*^6^, *Rhinella marina* (Bioproject: PRJEB24695, ID: 445546), *Xenopus laevis*^12^, and *Xenopus tropicalis*^13^. Furthermore, several conserved morphological characteristics shared by anurans make both taxonomic classification and phylogenetic analysis difficult to perform^14^. This stresses the need for combining novel genomic information with morphological and karyological data, as well as mitochondrial DNA sequencing, in order to improve accuracy in phylogenetic studies.

Next Generation Sequencing (NGS) provides a cost-effective and rapid method to sequence and analyse complete genomes. However, amphibians have a very high DNA content and a large proportion of repetitive and non-coding sequences^15^; thus, whole-genome assembly is still expensive and bioinformatically challenging. In contrast, high-throughput RNA-sequencing (RNA-Seq) is an affordable NGS technique that provides a convenient platform for transcript profiling and transcriptome sequencing in non-model amphibian species like *R. arenarum*^16,17^.

Here, we report for the first time the *de novo* assembly of *R. arenarum* transcriptome using massive RNA-Seq, followed by gene annotation and phylogenetic analysis.

## Results and Discussion

### Quality control, *de novo* assembly and transcriptome optimisation

**Figure 1:**
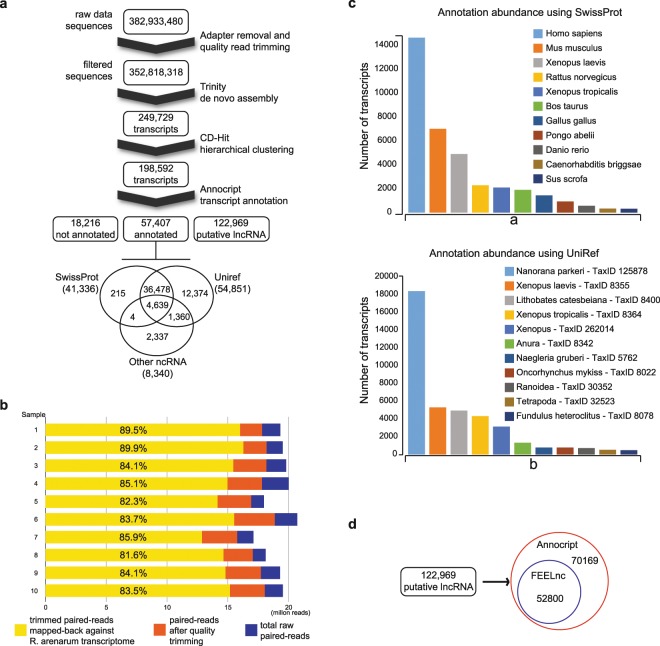
*De novo* transcriptome assembly of *Rhinella arenarum*. (**a**) Flow diagram of the assembly, from raw data to annotated transcripts. (**b**) For each of the ten samples, representation of total paired reads (blue), total paired reads after adapter removal and quality trimming (orange) and trimmed paired reads mapped-back against the *de novo* assembled transcriptome (yellow) are shown. (**c**) The number of transcripts annotated for the species present in reference databases (a: annotation using SwissProt DB; b: annotation using UniRef DB). (**d**) The number of lncRNAs defined by Annocript pipeline and confirmed by FEELnc tool.

As there is no reference genome for *R. arenarum*, we performed a *de novo* transcriptome assembly for the initial tadpole stage following the pipeline showed in Fig. 1a. Larvae at complete operculum stage (Stage 25, according to Del Conte and Sirlin^18^) were obtained from ten independent samples (see Material and Methods) and processed for bulk transcriptome sequencing. First, we evaluated the RNA-Seq quality profiles using the FASTQC tool (https://github.com/s-andrews/FastQC). Good quality reads of the different profiles are essential for obtaining a consistent transcriptome to be used in further analyses. Thus, a quality trimming step using Trimmomatic 0.38^19^ was performed to remove low quality bases and adapter sequences. Then, the profiles from the ten independent samples were pooled (Fig. 1b), yielding 382,933,480 raw sequences which assured excellent transcripts coverage and variety to generate a high-quality and complete assembly^20,21^. Low-quality bases with a Phred quality score below 30 were trimmed from both readings ends. Reads shorter than 45 bases were also discarded, yielding 352,818,318 filtered sequences. Next, the *R. arenarum* larval transcriptome was *de novo* assembled using Trinity software, version 2.8 (http://trinityrnaseq.sourceforge.net/) with default parameters^22^. *De novo* assemblies are more challenging to achieve in the absence of a reference genome, and entail an additional effort in the case of a polyploid organism due to the presence of subgenomes^23^. Although polyploidy is common in amphibians, *R. arenarum* is not a polyploid organism^14^. Nevertheless, Trinity software version 2.8 improves the handling of non-strand-specific RNA-Seq and high polymorphism containing transcriptomes. Hence, the number of filtered sequences obtained from our procedure assures a high-quality assembly of the *R. arenarum* transcriptome. A total of 176,409,159 paired reads were used in the *de novo* assembly, generating a total of 249,729 transcripts and 156,941 Trinity ‘genes’ from 244,861,075 assembled bases, with median and mean transcript lengths of 411 and 980 bp, respectively, 44.6% of GC content, and an N50 of 2,151 bp (Table 1). The annotation of Trinity ‘genes’ comes from the methodology used by this software to generate contigs, cluster them, and finally assign identifiers, i.e. ‘gene’ and ‘isoform’ to the constructed transcripts. These terms are valid under the definition of a gene as “the part of the genome that is active and transcribed”. Afterwards, it is necessary to continue with any annotation pipeline providing transcript identification such as coding sequences, rRNA and lncRNA.

**Table 1:** *De novo* transcriptome assembly statistics.

	Trinity statistics	CD-hit statistics
Total transcripts	249,729	198,592
Total Trinity ‘genes’	156,941	155,511
Mean transcripts length (bp)	980	802
Median transcripts length (bp)	411	367
N50	2151	1626
GC content (%)	44.63	44.48

A large proportion of reads mapping back to the assembly (i.e. above 60–70%) indicates proper quality sequence reconstruction and a representative transcriptome^22^. Using botwie2 v2.3.4.3^24^, we found that more than 80% of the reads were mapped back, as proper pairs in each profile, to the *R. arenarum de novo* assembled transcriptome (Fig. 1b). Next, we assessed the quality of the assembled transcriptome using TRANSRATE v1.0.3^25^, BUSCO v3.0.2^26^, and DETONATE v1.9^27^. These tools generate several metrics that serve as a guide to understand and evaluate error sources in the assembly process and provide evidence about the quality of the assembled transcriptome. We also assayed the assembled transcriptome through the hierarchical clustering tool CD-HIT, to address the possible generation of chimaeras, redundant transcripts and fragmented assemblies common to the process of *de novo* assembly^28,29^. Table 2 contains the principal scores obtained for the three analysis tools before and after CD-HIT processing (complete tables are provided in Supplementary Information as Table 1).

**Table 2:** Quality control of the *Rhinella arenarum* transcriptome. Quality scores were calculated using TRANSRATE v1.0.3, BUSCO v3.0.2, and DETONATE v1.9 before and after the CD-HIT clustering tool.

	Before CD-HIT	After CD-HIT
**TRANSRATE v1.0.3**		
Transrate Assembly Score	0.0457	0.158
Transrate Optimal Score	0.1172	0.2092
Transrate Optimal Cutoff	0.129	0.0928
good contigs	163674	173616
p good contigs	0.66	0.87
**BUSCO v3.0.2**		
Complete BUSCOs (C)	973 (99.4%)	972 (99.4%)
Complete and single-copy BUSCOs (S)	507 (51.8%)	740 (75.7%)
Complete and duplicated BUSCOs (D)	466 (47.6%)	232 (23.7%)
Fragmented BUSCOs (F)	0 (0.0%)	0 (0.0%)
Missing BUSCOs (M)	5 (0.6%)	6 (0.6%)
Total BUSCO groups searched	978	978
**DETONATE v1.9**		
Score	−14949658702	−15357930779
BIC_penalty	−2381204	−1893606,56
Prior_score_on_contig_lengths_(f_function_canceled)	−809045	−628519,94
Prior_score_on_contig_sequences	−339449528	−220853915,1
Data_likelihood_in_log_space_without_correction	−14607924788	−15135245313
Correction_term_(f_function_canceled)	−905861	−690575,74

To increase confidence in the quality and completeness of the optimised *R. arenarum* larval transcriptome, we performed a comparative interpretation through tentative orthologue assignments. BUSCO assessment, which estimates assembly quality based on evolutionarily-informed expectations of gene content from orthologues selected from OrthoDB v9^30^ (https://www.orthodb.org/), showed that the number of “Complete and single-copy” loci increased from 51.8% to 75.7% after CD-HIT processing. The assembled transcriptome included a total of 198,592 transcripts and 155,511 Trinity ‘genes’ with median and mean transcript lengths of 367 and 801 bp, respectively, 44.5% of GC content, and an N50 of 1626 bp (Table 1), representing 99.4% of completeness. Thus, CD-HIT sensibly improved transcriptome assembly, as evidenced by the reduction in the number of transcripts generated by Trinity (Table 1) and the improvement in quality scores (Table 2).

### Gene annotation

After quality evaluation and filtration, the assembled transcriptome was interrogated to obtain useful annotations for further analysis. This step was carried out using the pipeline Annocript^31^. With this tool, the *de novo* transcriptome was annotated by several BLAST analyses against UniProt and NCBI’s Conserved Domain Database and Nucleotide divisions. Besides, the pipeline added functional annotations for Gene Ontology terms, the Enzyme Commission classification, and Pathway databases. We also used Annocript to identify putative lncRNAs following four criteria: (*i)* non-annotation, lack of similarity with any protein, domain, or another ncRNA (any RNA species annotated in Rfam, including rRNAs); (*ii)* transcript length ≥ 200 nucleotides; (*iii)* an ORF < 100 amino acids; and (*iv)* non-coding potential score ≥ 0.95.

The statistics for the annotations obtained for the *R. arenarum* transcriptome are presented in Table 3. Out of 198,592 transcripts obtained after CD-HIT clustering, 18,216 could not be annotated and remain unidentified in available databases up to date (April 2019). Of the remaining transcripts, 57,407 (32.0%) could be annotated based on available information. Of these, 41,336 (72.0%) had hits for Swiss-Prot, 54,851 (95.5%) for UniRef, and 8,340 (14.5%) for non-coding RNAs consisting mainly of tRNAs and rRNAs (i.e. not lncRNAs). From this annotation, 36,478 transcripts (63.5%) shared annotations for SwissProt and UniRef, 4 for Swiss-Prot and ncRNA, 1,360 for UniRef and ncRNA, and 4,639 annotations were shared by the three databases. Besides, there were 215 unique matches for Swiss-Prot, 12,374 for UniRef, and 2,337 hits annotated as other ncRNAs (Fig. 1a and Table 3). Based on strand alignment analysis for Swiss-Prot annotations, we found that 19,546 and 21,790 transcripts were aligned to the positive and negative strands, respectively. For UniRef annotations, in turn, 26,166 and 28,685 transcripts were aligned to the positive and negative strands, respectively. Meanwhile, of 57,407 annotated transcripts, 44,760 (77.9%) were in agreement with the longest ORF. Finally, from the 44,760 longest ORF annotated transcripts, 17,423 (39.9%) were unique transcripts, and 27,337 (60.1%) were isoform transcripts.

**Table 3:** Statistics from Annocript annotation of the *Rhinella arenarum* transcriptome.

**Annotation statistics**	
Total number of sequences	198,592
Minimum sequence length	200
Maximum sequence length	22,320
Average percentage of Adenine	27.93
Average percentage of Guanine	21.71
Average percentage of Thymine	28.11
Average percentage of Cytosine	22.25
Average percentage of GC	44.48
**Number of blast results**	
Swiss-Prot	41,336
UniRef	54,851
Ribosomal RNAs	8,340
**Alignments in the positive strand**	
Swiss-Prot	19,546
UniRef	26,166
**Alignments in the negative strand**	
Swiss-Prot	21,790
UniRef	28,685
Transcripts with at least one blast result	57,407
Transcripts in agreement with the longest ORF	44,760
Unique transcripts	17,423
Isoform transcripts	27,337
Number of non-coding sequences	122,969
Number of non-annotated sequences	18,216

We also analysed which were the species closest to our annotated *de novo* transcriptome (Fig. 1c). On SwissProt, the top five species were *Homo sapiens, Mus musculus, Xenopus laevis, Rattus norvegicus*, and *Xenopus tropicalis*, which is not surprising because a high proportion of annotations in this database correspond to *H. sapiens* and *M. musculus* (Fig. 1c). However, on the curated multi-species database UniRef90, the top five closest species were *Nanorana parkeri* (TaxID 125878), *Xenopus laevis* (TaxID 8355), *Lithobates catesbeiana* (TaxID 8400), *Xenopus tropicalis* (TaxID 8364), and *Xenopus* (TaxID 262014), all belonging to the taxonomic class Amphibia (Fig. 1c).

The Annocript algorithm interpreted 122,969 transcripts as putative lncRNAs (Table 3), which were next tested using the FEELnc tool^32^. The latter uses a machine-learning method trained with coding transcripts to compute the coding potential score (CPS) for each transcript. The CPS maximises classification performances and infers whether a transcript is coding or non-coding in order to identify high-confidence sets of lncRNAs. From the 122,969 putative lncRNAs defined by Annocript, 52,800 transcripts (42.9%) were confirmed by FEELnc as a set of high-confidence lncRNA transcripts for *R. arenarum* (Fig. 1d and Supplementary File SF1). The 18,216 transcripts that could not be annotated, as well as the 70,169 putative lncRNAs not confirmed by FEELnc, may be artefacts or misassemblies inherent to the *de novo* assembly method^33,34^. Still, another reason why some lncRNAs could not be identified/annotated may be simply the lack of information in current databases. In this regard, it is worth noting that the identification/annotation of lncRNA is still complicated even for well-annotated species with fully sequenced reference genomes such as *H. sapiens* and *M. musculus*^35–37^.

The number of *R. arenarum* transcripts annotated as lncRNAs is higher than those reported for the amphibians *Xenopus tropicalis, Xenopus laevis* and *Lithobates catesbeiana*^6,38–40^. In the case of the transcriptome studies in *Xenopus*, the number of lncRNAs defined depends on the temporal and spatial expression profiling of the samples. In this sense, Necsulea *et al*.^41^ studied the evolutionary history of lncRNAs from polyadenylated transcriptomes of 8 organs and 11 species (human, chimpanzee, bonobo, gorilla, orangutan, macaque, mouse, opossum, platypus, chicken and frog) showing that lncRNAs are actively regulated and may function predominantly during the embryonic development. On the other hand, it is essential to note that the annotations obtained from genomes as predicted transcripts are always lower than the plethora and repertoire of transcripts identified in a transcriptome. In our case, lncRNA transcript assessment followed a strict depuration process starting with the four-criteria Annocript pipeline annotation, followed by FEELnc confidence maximisation. Thus, our lncRNA dataset provides the starting point for future lncRNA studies in *R. arenarum*, which would allow to verify them and to assess their regulation and function. Undoubtedly, the future availability of *R. arenarum* genome will help curate the lncRNA set using strategies like the identification of promotor regions followed by non-coding regions.

At the moment, no consensus pipeline can be defined as the best for transcriptome assembly^42^. The state-of-the-art of the *de novo* assembly of transcripts can be summarised in the selection of raw sequences with good quality, *de novo* assembly using an appropriate tool, quality validation of the assembly through different software tools and their subsequent availability to the scientific community. Once a set of sequences is released, different research groups making use of them perform its curation. In this sense, we selected some genes from the transcriptome that are of our research interest, designed primers and then sequenced the PCR products by Sanger method in order to validate them. Until now, we have evaluated nine genes using independent biological samples (in Supplementary Information as Table 2), and sequences have been confirmed by alignment against the transcriptome and by annotation comparison with RefSeq and UniProtKB databases. Future sequencing of the *R. arenarum* genome would represent a unique opportunity since RNA-Seq data could be used to curate the genome assembly and vice versa.

All transcript contigs of this Transcriptome Shotgun Assembly project have been deposited at DDBJ/EMBL/GenBank under the accession number GHCG00000000. The version described in this paper is the first version, GHCG01000000. To facilitate the obtention of gene sequences, we designed a webpage (available at http://rhinella.uncoma.edu.ar) in which the annotated transcriptome for this non-model species can be readily accessed. It allows searching by “gene name” or “name description”, to retrieve data related to the *R. arenarum* transcriptome. The information contains sequence annotation, nucleotide and peptide sequence data, orthologous information and annotated pathways.

### Phylogenetic analysis

To conduct phylogenetic studies with *R. arenarum* transcriptome data, we downloaded all the protein sequences annotated for the order Anura from UniProtKB taxonomy and constructed a matrix of 5423 anurans × 7376 proteins. Then, we filled the matrix for absent sequences (0; no protein sequence) or present sequences (1; protein sequence) for each anuran. We calculated the Jaccard distance^43^ using the Philentropy R package^44^ and selected a final cluster of 55 anurans and 28 protein sequences for each Anura (Supplementary File SF2). Next, we included in the set the corresponding 28 protein sequences derived from the *R. arenarum* transcriptome and performed multiple alignments for each protein using Muscle algorithm^45^, available in the MegaX software^46^. Finally, we concatenated the 28 alignments and applied the Maximum Parsimony method (1000 bootstraps) to construct the evolutionary history^47^.

**Figure 2:**
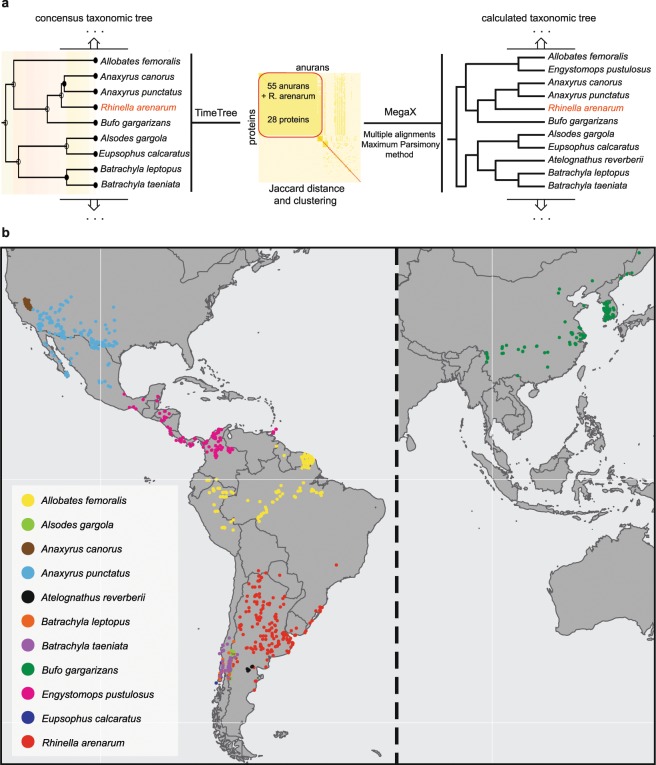
Phylogenetic analysis using *Rhinella arenarum* transcriptomic data. (**a**) Consensus taxonomic tree (TimeTree) and calculated taxonomic tree using 28 protein sequences for 55 anurans + *Rhinella arenarum*. (**b**) Geolocation of the anurans present in the same clade as *R. arenarum*.

Besides, we interrogated the TimeTree database^48^ with the 56 anurans (55 + *R. arenarum*) to obtain a consensus taxonomic tree. When we compared our experimental tree against the consensus tree, there were two main differences: first, *Engystomops pustulosus* clustered together with *Allobates femoralis* in our analysis, while in TimeTree *E. pustulosus* clustered in another clade (Supplementary Information – Fig. S1a,b); second, the species *Amietia lubrica, Atelognathus reverberii, Breviceps macrops, Callulops wilhelmanus, Cophixalus cheesmanae, Cornufer pelewensis, Craugastor fitzingeri*, and *Hyperolius bolifambae* are not present in the TimeTree database. Nevertheless, *R. arenarum* was included in the same clade in both trees (Fig. 2a).

Next, we retrieved biodiversity data from the 56 anurans using gbif R package^49^ and mapped the coordinates for each species on a world map using maps R package^50^ (Supplementary Information – Fig. S2). Figure 2b shows the anuran species present in the clades closer to *R. arenarum*. All the species are spread throughout the American continent, except for *Bufo gargarizans*, which is mainly found in eastern Asia. Since the gene sequence-based phylogenetic analysis of *R. arenarum* is consistent with the consensus taxonomic tree for this species, we conclude that the *R. arenarum* transcriptome is highly reliable to perform evolutionary studies, identify new subspecies, and further characterise subspecies like *Rhinella arenarum arenarum* and *Rhinella arenarum mendocinus*.

## Conclusions

We report for the first time a transcriptome for the non-model organism *Rhinella arenarum* at the first larvae (complete operculum) stage. The reconstructed transcriptome reached 99.4% of completeness and yielded a set of 57,407 well-annotated transcripts available for downstream analyses (http://rhinella.uncoma.edu.ar). Besides, a high confidence set of 52,800 putative lncRNAs was defined, and the feasibility of phylogenetic analyses was confirmed. The genomic tool delivered here will support biomarker assessment and discovery in ecology and toxicogenomics and facilitate evolutionary and global comparative genomic diversity analyses.

## Materials and Methods

### Experimental procedures

#### *R. arenarum* embryo development

Adult *R. arenarum* females and males were collected in a pristine environment at Los Barreales Lake (S38.45344 W68.72918) during the breeding (spring) season and maintained in an outdoor terrarium. All efforts were made to minimise the stress and suffering of animals according to the recommended standards of the American Society of Ichthyologists and Herpetologists (ASIH) in Guidelines for the Use of Live Amphibians and Reptiles in Field Research (http://www.asih.org/pubs/). An *ad hoc* Committee of the Centre for Research in Environmental Toxicology and Agrobiotechnology of Comahue (CITAAC, http://citaac.uncoma.edu.ar), which currently reviews and approves the projects that require the use of laboratory and field animals, approved the *Rhinella arenarum* project. Also, the collection guide of field specimens and their use in our projects were presented and approved by the Environment Bureau of the Province of Neuquén through the Applied Ecology Center of Neuquén, Argentina. Embryos were obtained by *in vitro* fertilisation and developed until complete operculum stage (Stage 25, according to Del Conte and Sirlin^18^) as described before^5^.

### RNA extraction, cDNA library generation and massively parallel sequencing

For deep transcriptome sequencing, *R*. arenarum larvae were grown in 10 different glass receptacles. From each receptacle, fifteen random larvae were randomly collected and pooled in a tube containing RNAlater® solution (Thermo Fisher Scientific Inc.) (15 larvae/1 tube/1 sample). Then, total RNA of each sample was extracted using the Thermo Scientific GeneJET RNA Purification Kit (Thermo Fisher Scientific Inc.). RNA quantity and quality were evaluated using Agilent RNA 6000 Pico Kit in a 2100 Bioanalyzer (Agilent Technologies). All samples presented an optimal condition [RNA Integrity Number (RIN) > 8]^51^. The cDNA library for transcriptome analysis was prepared using the TruSeq® RNA v2 kit (Illumina, San Diego, CA, USA). Briefly, mRNA enrichment was carried out from 1 µg of total RNA using oligo dT magnetic beads, followed by chemical fragmentation of the purified mRNA into small pieces, double-stranded cDNA synthesis, end repair, and adenylation processes. Finally, adaptor ligation and enrichment were carried out by PCR. The ten library samples were normalised to 10 nM cDNA and were sequenced in a line of Hiseq. 1500 Illumina, generating non-strand specific “paired-ends” (PE) 2 × 100 bp readings.

## Supplementary information

Supplementary Information.

Dataset 1.

Dataset 2.

## Data Availability

Raw sequencing data have been deposited in the Bioproject PRJNA485066 in the NCBI repository.
