# Supplementary material for: The Rhinella arenarum transcriptome: de novo assembly, annotation and gene prediction
Source: Sci Rep. 2020 Jan 23;10:1053. doi: 10.1038/s41598-020-57961-4 (PMC6978513; doi:10.1038/s41598-020-57961-4)
Supplement: Supplementary file 1 — Supplementary Information. [file 41598_2020_57961_MOESM1_ESM.pdf]

The *Rhinella arenarum* transcriptome: de novo assembly, annotation and gene prediction.

Danilo Guillermo Ceschin*, Natalia Susana Pires, Mariana Noelia Mardirosian, Cecilia Inés Lascano and Andrés Venturino

*Corresponding author: danilo.ceschin@iucbc.edu.ar

Supplementary Information

A.

DETONATE v1.9	Before CD-HIT	After CD-HIT
Score	-14949658702.30	-15357930778.64
BIC_penalty	-2381203.60	-1893606.56
Prior_score_on_contig_lengths_(f_function_canceled)	-809044.58	-628519.94
Prior_score_on_contig_sequences	-339449527.53	-220853915.05
Data_likelihood_in_log_space_without_correction	-14607924787.84	-15135245312.83
Correction_term_(f_function_canceled)	-905861.26	-690575.74
Number_of_contigs	249729	198592
Expected_number_of_aligned_reads_given_the_data	155643831.69	153902600.16
Number_of_contigs_smaller_than_expected_read/fragment_length	0	0
Number_of_contigs_with_no_read_aligned_to	13365	5759
Maximum_data_likelihood_in_log_space	-14606640754.41	-15134046485.73
Number_of_alignable_reads	158982726	157741943
Number_of_alignments_in_total	479349679	311193782

B.

BUSCO v3.0.2	Before CD-HIT	After CD-HIT
Complete BUSCOs (C)	973 (99.4%)	972 (99.4%)
Complete and single-copy BUSCOs (S)	507 (51.8%)	740 (75.7%)
Complete and duplicated BUSCOs (D)	466 (47.6%)	232 (23.7%)
Fragmented BUSCOs (F)	0 (0.0%)	0 (0.0%)
Missing BUSCOs (M)	5 (0.6%)	6 (0.6%)
Total BUSCO groups searched	978	978

C.

TRANSRATE v1.0.3	Before CD-HIT	After CD-HIT
n seqs	249729	198592
smallest	177	181
largest	22320	22320
n bases	244861075	159312424
mean len	980.46	802.18
n under 200	56	26
n over 1k	64912	39192
n over 10k	492	244
n with orf	50548	31484
mean orf percent	50.5	51.09
n90	328	288
n70	1021	667
n50	2152	1626
n30	3564	3013
n10	6316	5709
gc	0.45	0.44
bases n	0	0
proportion n	0.0	0.0
**Read mapping metrics:**		
fragments	191466740	191466740
fragments mapped	63348148	103451452
p fragments mapped	0.33	0.54
good mappings	55016276	89993226
p good mapping	0.29	0.47
bad mappings	8331872	13458226
potential bridges	0	0
bases uncovered	159352468	55577091
p bases uncovered	0.65	0.35
contigs uncovbase	164960	105270
p contigs uncovbase	0.66	0.53
contigs uncovered	249729	198592
p contigs uncovered	1.0	1.0
contigs lowcovered	249729	198592
p contigs lowcovered	1.0	1.0
contigs segmented	21546	20408
p contigs segmented	0.09	0.1
TRANSRATE ASSEMBLY SCORE	0.0457	0.1588
TRANSRATE OPTIMAL SCORE	0.1172	0.2092
TRANSRATE OPTIMAL CUTOFF	0.129	0.0925
good contigs	163674	173616
p good contigs	0.66	0.87

Supplementary Table 1. Quality scores for the *Rhinella arenarum* transcriptome. Complete tables for the tools used to evaluate the quality of the *de novo* transcriptome assembly, before and after CD-HIT clustering: A. DETONATE v1.9; B. BUSCO v3.0.2 and C. TRANSRATE v1.0.3.

Gene	Description
ODC1	Ornithine Decarboxylase 1
ACTB	Actin Beta
JUN	Jun Proto-Oncogene, AP-1 Transcription Factor Subunit
APAO	Acetylpolyamine Oxidase
SOD1	Superoxide Dismutase 1
DAO	Diamine Oxidase
AMD1	Adenosylmethionine Decarboxylase 1
FOS	Fos Proto-Oncogene, AP-1 Transcription Factor Subunit
RPL8	Ribosomal Protein L8

Supplementary Table 2. Transcriptome genes validated by Sanger sequencing.

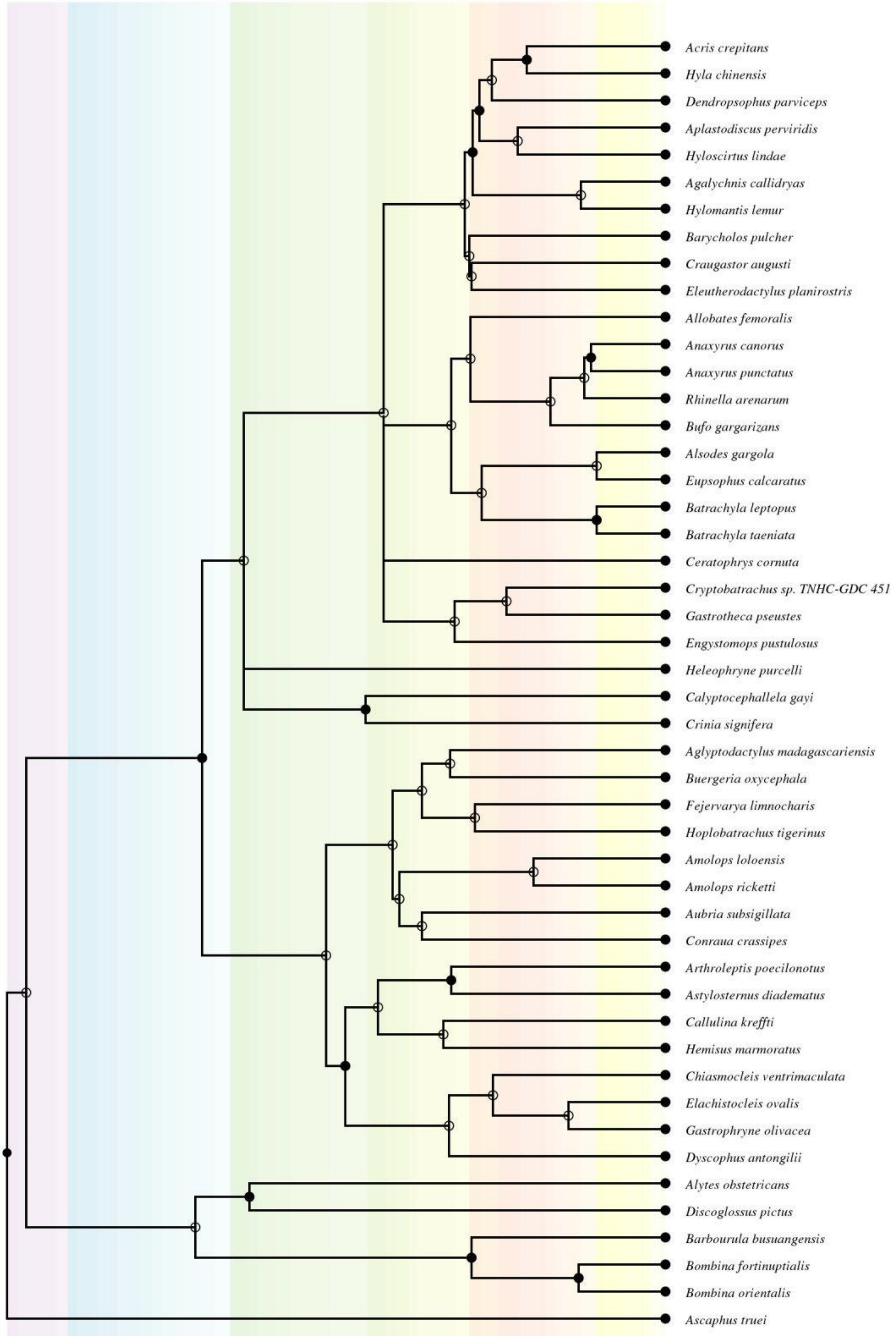

Figure S1-a. TimeTree consensus taxonomic tree

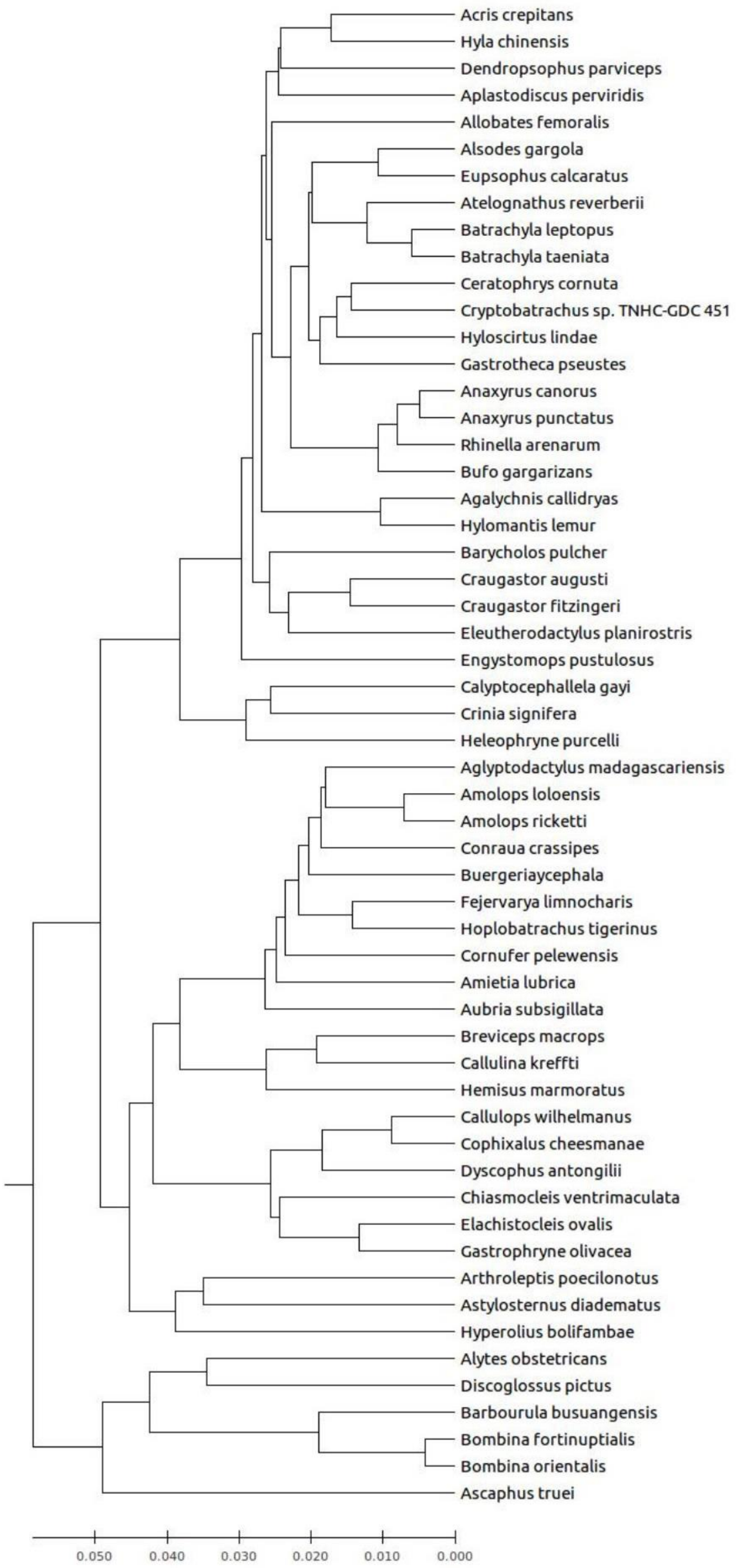

Figure S1-b. Calculated taxonomic tree

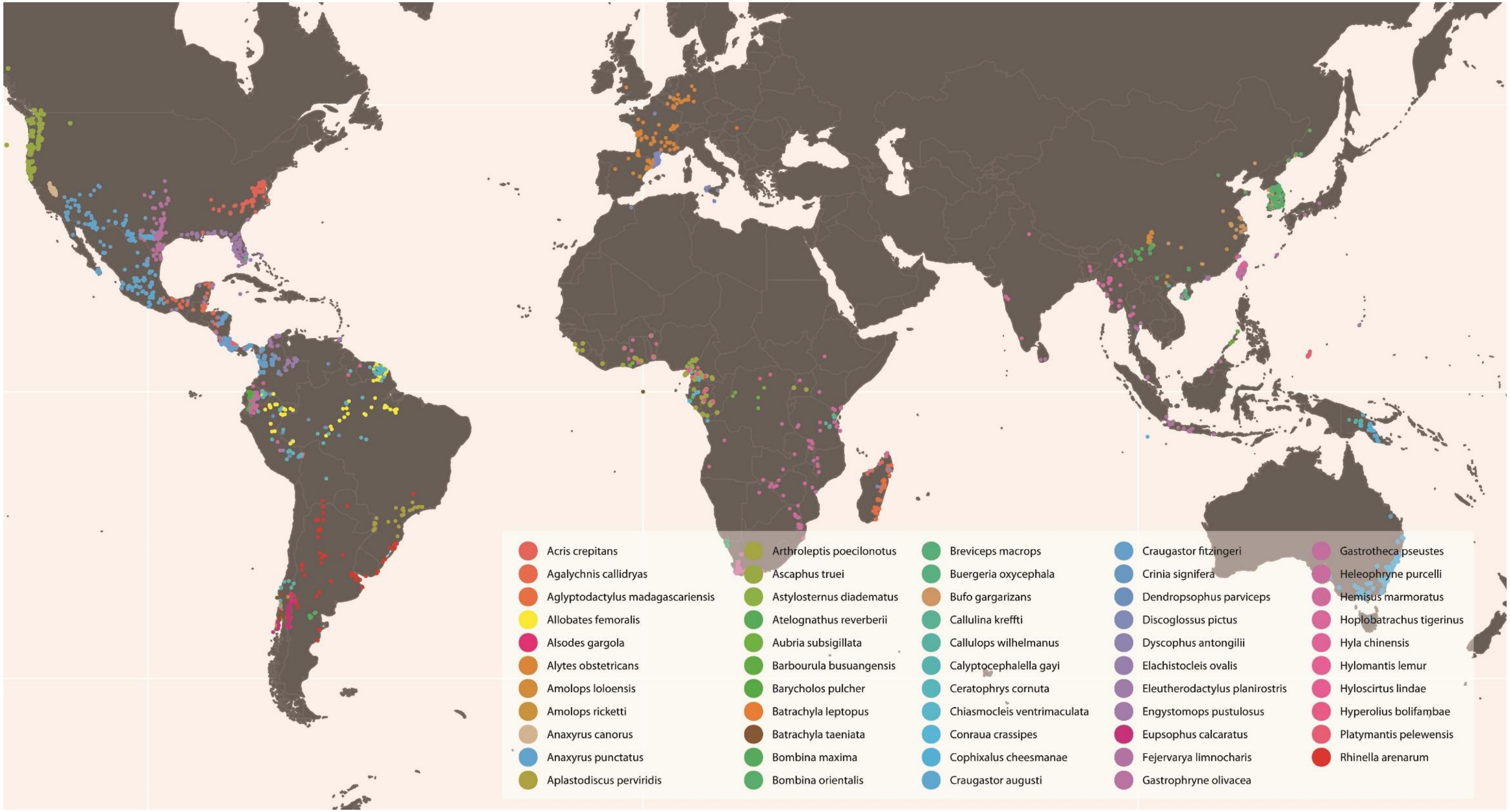

Figure S2. Geolocalization of the 56 anura species used for the phylogenetic analysis.

.
